# The Chromatin Remodeling Factor CHD5 Is a Transcriptional Repressor of *WEE1*

**DOI:** 10.1371/journal.pone.0108066

**Published:** 2014-09-23

**Authors:** Jinhua Quan, Guillaume Adelmant, Jarrod A. Marto, A. Thomas Look, Timur Yusufzai

**Affiliations:** 1 Department of Radiation Oncology, Dana-Farber Cancer Institute, Boston, Massachusetts, United States of America; 2 Department of Biological Chemistry & Molecular Pharmacology, Harvard Medical School, Boston, Massachusetts, United States of America; 3 Blais Proteomics Center, Department of Cancer Biology, Dana-Farber Cancer Institute, Boston, Massachusetts, United States of America; 4 Department of Pediatric Oncology, Dana-Farber Cancer Institute, Boston, Massachusetts, United States of America; Bellvitge Biomedical Research Institute (IDIBELL), Spain

## Abstract

Loss of the chromatin remodeling ATPase CHD5 has been linked to the progression of neuroblastoma tumors, yet the underlying mechanisms behind the tumor suppressor role of CHD5 are unknown. In this study, we purified the human CHD5 complex and found that CHD5 is a component of the full NuRD transcriptional repressor complex, which also contains methyl-CpG binding proteins and histone deacetylases. The CHD5/NuRD complex appears mutually exclusive with the related CHD4/NuRD complex as overexpression of CHD5 results in loss of the CHD4 protein in cells. Following a search for genes that are regulated by CHD5 in neuroblastoma cells, we found that CHD5 binds to and represses the G2/M checkpoint gene *WEE1*. Reintroduction of CHD5 into neuroblastoma cells represses *WEE1* expression, demonstrating that CHD5 can function as a repressor in cells. A catalytically inactive mutant version of CHD5 is able to associate with a NuRD cofactor but fails to repress transcription. Our study shows that CHD5 is a NuRD-associated transcriptional repressor and identifies *WEE1* as one of the CHD5-regulated genes that may link CHD5 to tumor suppression.

## Introduction

Chromodomain helicase DNA binding protein 5 (CHD5) is a chromatin remodeling factor and a member of the SNF2-like family of ATPases [1]–[4]. CHD5 is preferentially expressed in the vertebrate nervous system and disruption of its expression levels leads to neural tube defects in mice [5], [6]. In adults, expression of CHD5 is frequently lost in a variety of cancers either due to deletions of chromosome 1p36.3 [7]–[9] or due to CHD5 promoter CpG-hypermethylation [10], [11]. Loss of CHD5 is correlated with a poor prognosis in neuroblastoma tumors, and neuroblastoma cells that express CHD5 are more responsive to treatment than those that do not express CHD5 [12]. Similarly, low expression of CHD5 in pancreatic cancers correlates with lower survival following chemotherapy [13].

While the links between CHD5 and cancer progression are rapidly emerging, the role of CHD5 in cells remains poorly understood. CHD5 is most closely related to the widely expressed chromatin remodeling ATPase CHD4 (also known as Mi-2beta), which is a subunit of the Nucleosome Remodeling and Deacetylase (NuRD) complex [14]–[17]. The NuRD complex contains two catalytic activities, ATP-dependent chromatin remodeling and histone deacetylation, and is generally involved in repressing transcription (reviewed in [18]). The CHD4-containing NuRD complex consists of the CHD4 ATPase, the histone deacetylases HDAC1 and 2, the histone-binding proteins RbAP46/48, the methyl-CpG-binding domain proteins MBD2 or MBD3, p66a and p66b (also known as GATAD2A and GATAD2B), and the metastasis-associated (MTA) proteins MTA1, MTA2, and MTA3. While the precise distribution of subunits across NuRD complexes is not well understood, multiple complexes have been shown to exist that contain some combination of the core subunits. For example, NuRD complexes have been isolated that contain either MBD2 or MBD3 but not both [19].

In contrast to CHD4, little is known about the interacting partners and cellular role of CHD5. CHD5 from rats has been shown to interact with some subunits of the NuRD complex, suggesting that CHD5 may also be part of a NuRD-like complex and, therefore, repress transcription [20]. In addition, CHD5 was reported to be enriched at promoters of specific genes, further supporting a role for CHD5 in transcriptional regulation [5], [20], [21]. Whether CHD5 is part of a full NuRD complex in human cells and whether its ATPase activity is required to regulate transcription has not been shown. It is also unclear why cells of the sympathetic nervous system express both CHD4 and CHD5, but recent evidence indicates that these proteins may play distinct functions. A study characterizing the chromatin remodeling activity of CHD5 found it remodels nucleosomes through a distinct unwrapping activity that is not readily observed for CHD4 [4]. Whether the differences in the cellular roles of CHD4 and CHD5 are due to differences in the manner that CHD4 and CHD5 remodel nucleosomes or whether they reflect other, unknown features of the two ATPases is still not known. In this study, we found that CHD5 interacts with the full NuRD complex in human cells and appears to compete with CHD4 for incorporation into the complex. We also found that CHD5 represses transcription of *WEE1*, in both neuroblastoma and pancreatic cancer cell lines. Moreover, the CHD5 protein can be detected at the *WEE1* promoter in pancreatic cancer cells that express endogenous CHD5. Finally, a mutant version of CHD5 that is unable to remodel chromatin can associate with other NuRD subunits but does not fully repress *WEE1* transcription. In summary, our study shows that human CHD5 functions as a bona fide transcriptional repressor and identifies a CHD5-dependent transcription pathway in cancer cells.

## Materials and Methods

### Analysis of the CHD5/NuRD complex

The cDNAs of CHD5 (Open Biosystems) and CHD4 (DF/HCC DNA Resource Core) were subcloned into pOZ-N, which expresses the cDNAs as FLAG/HA N-terminal fusions [22]. HeLa S3 cell lines that stably express pOZ-N-CHD5 were generated, and CHD5 complexes were purified by tandem affinity purification as described [22]–[24]. The purified material was analyzed by mass spectrometry at the Blais Proteomics Center at the Dana-Farber Cancer Institute in order to identify co-purifying factors. The list of potential binding partners was filtered to remove proteins identified with a frequency>1% across a large set of negative control TAPs. In addition, we required that at least one peptide of the reported protein partner could be matched unambiguously to a single gene [25].

Protein interactions were confirmed by anti-FLAG co-immunoprecipitation followed by western blotting. HEK293T-derived Phoenix A cells grown in 10 cm dishes were transiently transfected by CaPO4 [22] with the empty pOZ-N vector, or pOZ-N containing the human cDNA for CHD4 (pOZ-N-CHD4), wild-type CHD5 (pOZ-N-CHD5), or mutant CHD5 (pOZ-N-mutCHD5). Two days after transfection, the cells were collected, washed with 1X PBS, and lysed in Lysis Buffer [20 mM Tris-Cl, pH 7.6, 0.15 M NaCl, 1.5 mM MgCl2, 0.2 mM EDTA, 1% Triton X100, 1 mM DTT, 0.2 mM PMSF, 0.5 mM benzamidine]. The insoluble material was pelleted by centrifugation, and the extract was pre-cleared by incubation with IgG Sepharose 6 (GE Lifesciences) for 1 hour followed by a brief centrifugation to remove the resin. A sample of the extract was saved and the remainder of the extract was incubated with anti-FLAG resin (Sigma) for 3 hours. The resin was pelleted and washed three times with Lysis Buffer, and the proteins eluted in NuPage Sample Buffer (Life Technologies). An aliquot of the initial extract and the immunoprecipitated samples were resolved by SDS-PAGE. Western blotting was carried out using the following commercial antibodies: anti-CHD4 (SAB4200107; Sigma), anti-CHD5 (sc-271248; Santa Cruz Biotech.), anti-MTA2 (PA1-41581, Thermo Sci.), anti-HDAC1 (sc-7872, Santa Cruz Biotech.), anti-RbAP46/48 (4633, Cell Signaling Tech.), anti-MBD3 (sc-9402, Santa Cruz Biotech.), and anti-histone H3 (4499, Cell Signaling Tech.).

Nuclear and pellet extracts were prepared as previously described [26], [27] from PANC-1 cells that stably express pOZ-N-CHD5 or pOZ-N-mutCHD5. Briefly, isolated nuclei were resuspended and incubated for 30 min in a high-salt buffer containing 20 mM Hepes-K+, pH 7.6, 0.42 M KCl, 1.5 mM MgCl2, 20% glycerol, 0.2 mM EDTA, 1 mM DTT, and then centrifuged at 16000×g for 15 min to pellet the insoluble material. The pellet was resuspended by Dounce homogenization in a buffer containing 20 mM HEPES-K+, pH 7.6, 5 mM MgCl2, 20% glycerol, 0.2 mM EDTA, 1 mM DTT, and 1 mM CaCl2. The extract was then incubated with MNase (1 u/µl; Worthington) for 30 min at room temp, and centrifuged at 16000×g to remove any remaining insoluble material. The supernatant was collected and the volume adjusted so that the final volumes of the soluble and pellet extracts were the same. An equal aliquot was used in western blotting with the indicated antibodies and scanned using an Odyssey scanner (LiCor).

### Chromatin immunoprecipitation (ChIP)

Anti-CHD5 rabbit polyclonal antibodies were generated against a bacterially-expressed, recombinant fragment of CHD5 that is not conserved with other remodeling factors (Covance Research). Crosslinked chromatin was made by treating PANC-1 [35] and KELLY [34] cells with 1% formaldehyde for ∼30 min followed by nuclei extraction and sonication [30]. A sample of the soluble, crosslinked chromatin was uncrosslinked, deproteinized and resolved by agarose gel electrophoresis. Average DNA fragments were ∼700 bp. The concentration of the soluble chromatin was estimate by A260 and 140 ug of chromatin was used per IP with 40 µg of anti-CHD5 serum, pre-immune serum or anti-pol II antibodies (05-952, Millipore). The antibody-bound fragments were precipitated, uncrosslinked, and the DNA fragments purified as described [28]. Quantitative PCR of the *WEE1* locus or distal sites were performed in quadruplicate reactions using a SYBR green mix (Thermo). The primers used for the ChIP analyses are listed in Table S1. The ChIP assays were each performed at least three independent times. The enrichment values were calculated using the 2-ddCt comparing bound v. input or bound (antibody) v. bound (pre-immune) with normalization to a distal intergenic locus of chromosome 1.

### Quantitative RT-PCR

HEK293T-derived Phoenix A cells were grown in 10 cm dishes in DMEM (with 10% FBS/1% pen-strep) and transiently transfected by CaPO4. For the assay to monitor the levels of CHD4 protein, 0, 2, 5, 10, or 15 µg of pOZ-N-CHD5 DNA were transfected, and the empty pOZ-N plasmid was co-transfected (15, 13, 10, 5, 0 µg, respectively) so that the total amount of DNA in each transfection totaled 15 µg. After two days, the cells were harvested and split into two. Half of the cells were used to make whole cell extracts for western blotting (as described above) and the other half of the cells were used to extract total RNA using the RNeasy kit (Qiagen) with a DNaseI digestion step. For the assays used to monitor *WEE1* expression, we transfected approximately 0, 5, and 15 µg of the pOZ-N-CHD5 with co-transfection of the empty pOZ-N plasmid as described above. The KELLY cells were grown in RPMI (with 10% FBS/1% pen-strep) and transfected with 15 µg of pOZ-N-CHD5 using Lipofectamine 2000 (Life Technologies). The transfection efficiency of the KELLY cells varied, and was monitored by parallel transfection assays using a beta-gal reporter. PANC1 cells were grown in DMEM (with 10% FBS/1% pen-strep) and transfected with a scrambled, nontargeting control siRNA (IDT) or siRNAs that target human CHD5 [set 2:GCAUGUCAACGGGAAGUACAGCACC; set 6:AGUGUAAAGGGAAGCGGAAGAAGAA (IDT)].

The microarray analysis of KELLY cells transiently transfected with pOZ-N or pOZ-N-CHD5 was performed at the DFCI Microarray Core Facility using Human Gene 1.0 ST Arrays (Affymetrix). The raw data have been deposited and are publically available at NCBI [GEO Accession: GSE59899].

The RT-PCR reactions were carried out using 1 ug of total RNA with M-MuLV reverse-transcriptase (New England Biolabs) and SYBR FAST qPCR mix (Kapa Biosystems). The primer-sets to detect CHD5 transcripts were: Forward 5’- TGCTTAAAGGAGCCCAAGTC, Reverse 5’-TTGGTCAGCGTGTGGTAATC. The CHD4 primer-sets were based on those previously published (CHD4 set 1 in [29]). Standard curves were performed using these two primer-sets with CHD4 and CHD5 cDNAs to ensure comparable amplification efficiencies and no cross-amplification. The 5S rDNA transcript was used as an internal control with primer-sets previously reported [30]. The values from the quantitative RT-PCR assays were compared using the 2∧-ddCT method.

For the cell viability assay, KELLY cells were transfected with pOZ-N or pOZ-N-CHD5. Two days after transfection, the cells were counted and re-plated (Day 0) in media containing 2 mM of a WEE1 inhibitor (Cat: 681641, EMD Millipore) for an additional two days. The media was changed to fresh media with inhibitor and the cell viability was estimated on the indicated days using CellTiter-Glo (Promega).

## Results and Discussion

### Human CHD5 is a subunit of the full NuRD complex

**Figure 1:**
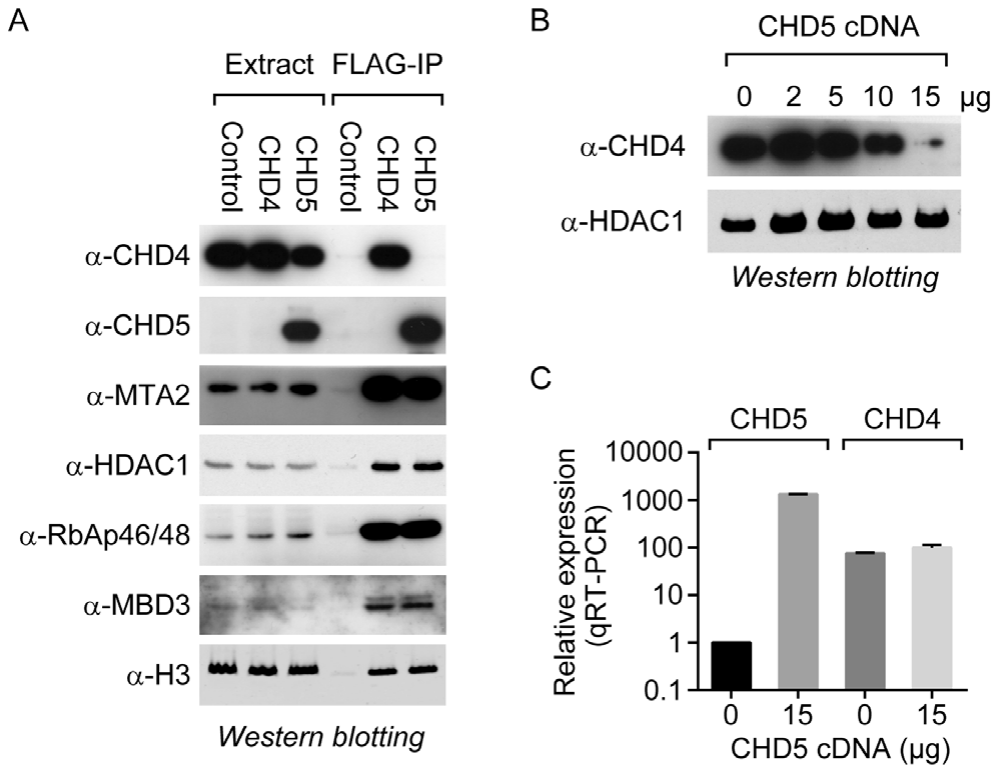
CHD5 co-purifies with the NuRD transcriptional repressor complex. (A) FLAG-tagged CHD4 and CHD5 were immunoprecipitated from a transiently transfected HEK293T-derived cell line, and the samples were analyzed by western blotting using the indicated antibodies. The empty vector was also transfected in parallel [Control]. (B) The HEK293T-derived line was transiently transfected with increasing amounts of the CHD5-expression plasmid. An empty vector was included where necessary to keep the final amount (15 µg) of transfected plasmid constant. Following transfection, cell extracts and RNA were prepared to measure CHD4 levels by western blotting and CHD4 and CHD5 mRNA levels by qRT-PCR.

To explore the role of CHD5 in cells, we purified the native CHD5-containing complex from a human cell-line and identified the binding partners of CHD5. For this analysis, we established a HeLa S3 cell line that stably expresses a FLAG/HA-tagged copy of human CHD5 and purified the CHD5 complex by tandem affinity purification. We then identified the CHD5-associated proteins by mass spectrometry [22], [31], [32]. Enrichment analysis [33] revealed that the set of 122 proteins that specifically co-purify with CHD5 is strongly enriched for GO-terms associated with the NuRD complex (log odds ratio 2.4, P_adj_<0.001). We found that the core members of the NuRD transcriptional repressor complex, with the exception of CHD4, were present in the FLAG/HA-CHD5 complex, including MTA1, MTA2, MTA3, HDAC1, HDAC2, MBD2, MBD3, RbAP46/48, and p66a/b (Figure S1). These results are consistent with the findings of Potts et al. [20], but also suggest that CHD5 and CHD4 might associate with NuRD in a mutually exclusive manner. To confirm the interaction between CHD5 and the subunits of the NuRD complex, we transiently transfected a HEK293T-derived cell line, which expresses endogenous CHD4, with plasmids expressing either FLAG-tagged CHD5 or FLAG-tagged CHD4. Following transfection, the epitope-tagged proteins were immunoprecipitated from whole-cell extracts, and the co-precipitating factors were analyzed by western blotting (Figure 1A). We found that both CHD4 and CHD5 interact with the same set of NuRD subunits but do not associate with each other, as no CHD4 was detected in the CHD5-containing complex isolated from HeLa cells or following immunoprecipitation of CHD5 from transiently transfected HEK293T-derived cells.

The finding that both CHD5 and CHD4 interact with the same set of cofactors suggests that these two enzymes might compete for interaction with the complex in cells that express both proteins. To test this hypothesis, we overexpressed CHD5 by transiently transfecting HEK293T-derived cells with increasing amounts of a CHD5 expression plasmid or an empty vector, and monitored the levels of CHD4 protein by western blotting. We found that overexpression of CHD5 results in an almost complete loss of CHD4 protein in cells, while the levels of HDAC1, a core member of the NuRD complex, remain unaltered (Figure 1B). Thus, the NuRD complexes in these cells appear to convert from CHD4-containing to CHD5-containing. To determine whether the reduction in the levels of CHD4 protein following overexpression of CHD5 is due to silencing of the *CHD4* gene, we measured the *CHD4* mRNA levels by quantitative RT-PCR. Following transfection of the CHD5 cDNA, we found that the levels of *CHD4* mRNA increased slightly, indicating the reduction in CHD4 is not due to inhibition of *CHD4* transcription (Figure 1C). We believe that the loss of CHD4 observed upon CHD5 overexpression is due to the competition between CHD4 and CHD5 for binding to the NuRD complex, which could result in exclusion of CHD4 from the complex, leading to its instability.

### CHD5 represses transcription of *WEE1*

**Figure 2:**
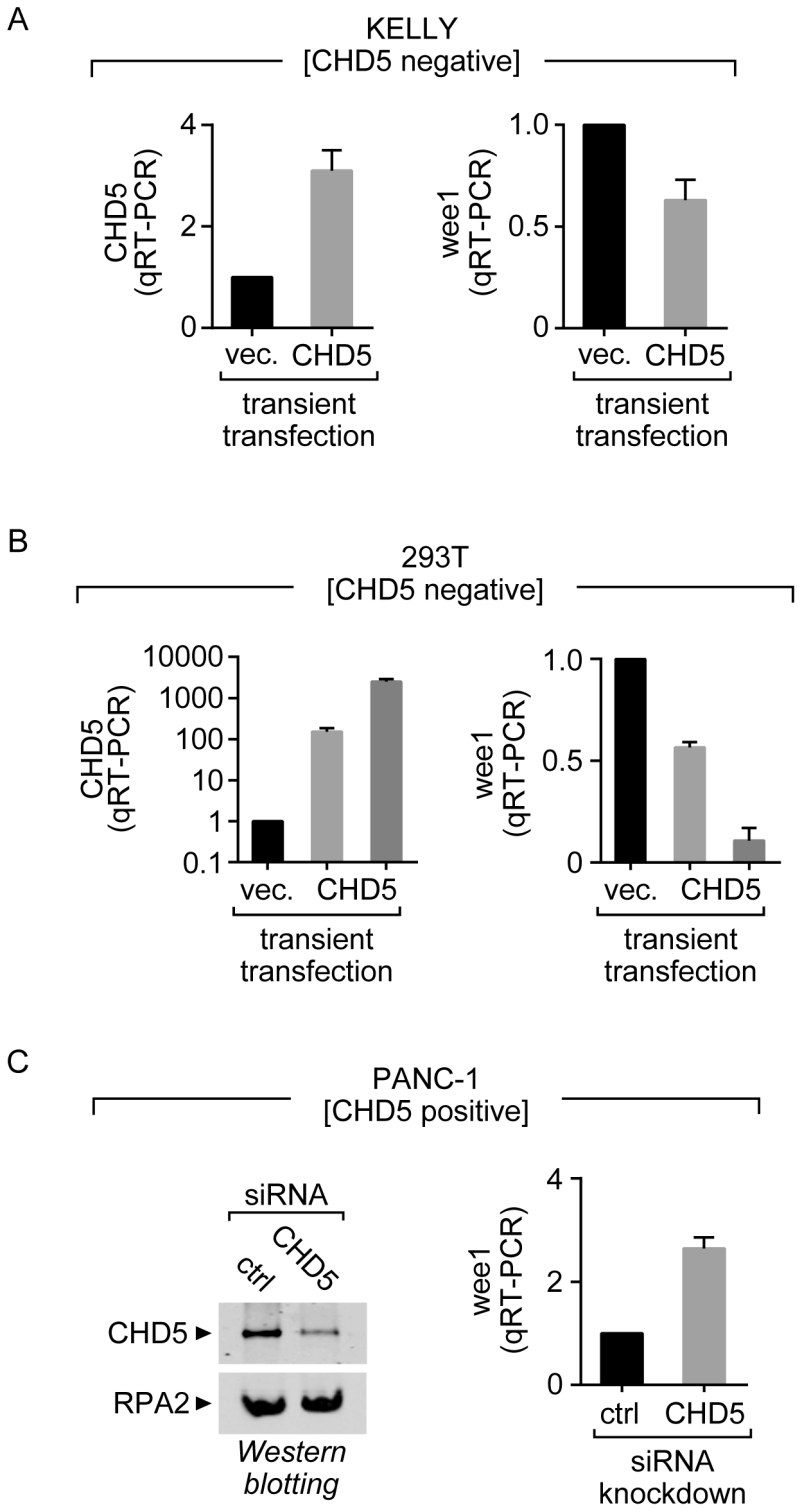
CHD5 expression leads to repression of *WEE1*. (A) The neuroblastoma line KELLY or (B) 293T-derived cells were transiently transfected with vector (vec.) or the vector containing the human CHD5 cDNA. In the case of the 293T-derived cells, two amounts of CHD5 cDNA were transfected (see Methods). Two days after transfection, the cells were harvested and total RNA was prepared. Quantitative RT-PCR was performed to monitor the levels of the *CHD5* and *WEE1* transcripts. The values shown are normalized to a 5S rRNA housekeeping transcript. (C) CHD5 levels were reduced siRNA-mediated knockdown in PANC-1 cells. As a control (ctrl), a scramble siRNA was transfected in parallel. The levels of CHD5 protein were detected by western blotting (left) and the levels of WEE1 mRNA were analyzed by qRT-PCR. Error bars represent SD [n = 3].

The interaction of CHD5 with the NuRD complex suggests that CHD5 might be involved in transcriptional repression. It also raises the possibility that loss of CHD5 during neuroblastoma progression disrupts specific transcriptional pathways involved in cell growth. To identify a candidate gene regulated by CHD5, we reintroduced CHD5 in a neuroblastoma cell line and performed pilot microarray analyses. For this study we chose KELLY cells [34], which we found do not express CHD5 and have a higher efficiency of transfection (<30%) when compared with other neuroblastoma cell lines. We transfected KELLY cells with a control vector or a vector containing the human CHD5 cDNA and, two days after transfection, compared the expression levels of multiple genes using microarrays. The raw data have been deposited at NCBI [GEO Accession: GSE59899]. We then identified the genes that showed changes in their expression levels following CHD5 expression. We found that the cell-cycle regulator *WEE1* was one of the genes that showed the most significant change in expression following the transfection of CHD5 (Figure S2). To validate this result, we repeated the transient transfection experiments and performed quantitative RT-PCR (qRT-PCR) using primers that amplify the *WEE1* mRNA or, as a control, the 5S rRNA house-keeping gene. As observed in the microarray analysis, the levels of the *WEE1* transcript consistently decrease by 30–40% following reintroduction of CHD5 (Figure 2A). We believe that the reduction in WEE1 transcription might have been even higher if the transfection efficiency of KELLY cells could be improved. It should also be noted that under our transfection conditions, the mRNA and protein levels of CHD4 do not change significantly, suggesting that expression of CHD5 in these cells is not sufficient to destabilize CHD4 (Figure S3A).

To further confirm that CHD5 expression leads to a decrease in *WEE1* mRNA, we repeated the transient transfection experiments using the HEK293T-derived cell line, which does not express CHD5 and has a higher efficiency of transfection than the KELLY cells. We transfected two different amounts of the CHD5 expressing plasmid into the cells and observed that the levels of CHD5 transcripts increased as more plasmid was transfected. We then analyzed the levels of *WEE1* transcripts and found that transfection of the CHD5-expressing plasmid reduced the levels of *WEE1* transcript. Further, the levels of *WEE1* transcripts in cells with high levels of CHD5 expression drop to approximately 10% of control transfected cells (Figure 2B). The almost complete loss of *WEE1* transcripts was only observed when we obtained very high transfection efficiencies, as lower levels of CHD5 transfection repress *WEE1* expression to a lesser degree. We should note that we cannot rule out the possibility that high levels of CHD5 expression could lead to other off-target effects that may contribute to the loss of *WEE1* expression. Nonetheless, using two cell lines, which do not have detectable levels of endogenously expressed CHD5, we have found that reintroduction of CHD5 leads to transcriptional repression of the candidate gene *WEE1*.

Next, we decided to further explore the connection between *WEE1* and CHD5 in cells that express CHD5 endogenously. For this analysis, we used the pancreatic cancer cell line PANC-1 [35], which expresses CHD5 at levels readily detectable by qRT-PCR and western blotting. We performed siRNA knockdown experiments to reduce the levels of CHD5 in these cells and then compared the levels of *WEE1* transcripts in the CHD5-knockdown cells to those in cells treated with a scrambled, control siRNA. We found that reducing the levels of CHD5 in the PANC-1 cells leads to a significant increase in *WEE1* transcripts (Figure 2C). It should be noted that our transient knockdown of CHD5 does not significantly affect the levels of CHD4 protein (Figure S3B). Taken together, our experiments, which use three different cell types, have shown that human CHD5 can act as a transcriptional repressor and that *WEE1* is one its target genes.

The WEE1 protein is an integral component of the G2/M checkpoint regulatory network. In normal cells, WEE1 is a ser/thr kinase that phosphorylates cyclin-dependent kinase 1 (CDK1; reviewed in [36]). Phosphorylation of CDK1 inhibits its activity and blocks entry into mitosis. It has been reported that some cancer cells, including neuroblastoma, have elevated levels of the WEE1 protein [37], [38]. The mechanism that ties *WEE1* expression to cancer growth is not well understood, although it has been suggested that WEE1 can act as an oncogene by blocking DNA damage-induced apoptosis [39]. In the case of neuroblastoma tumors, maintaining high levels of WEE1 is essential for survival, as studies have shown that neuroblastoma cells are sensitive to inhibitors of WEE1 [38].

### CHD5 is bound to the *WEE1* promoter

**Figure 3:**
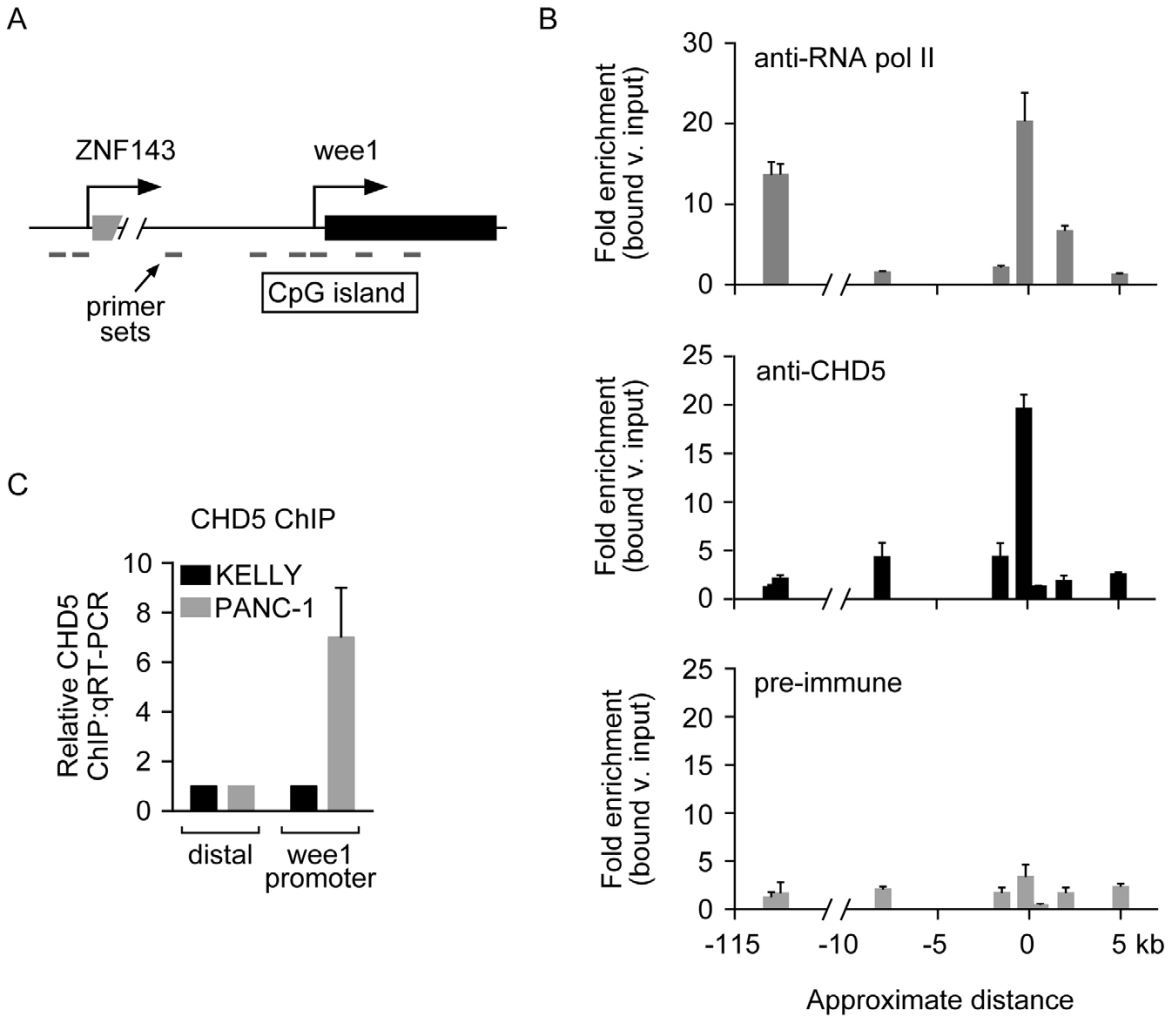
CHD5 is enriched at the *WEE1* promoter. (A) Schematic of the *WEE1* locus with the putative *WEE1* promoter and direction of transcription indicated. The promoter of the ZNF143 gene is approximately 115 kb upstream of *WEE1*. The horizontal bars depict the location of primersets used. (B) ChIP experiments using anti-RNA pol II antibodies show enrichment of RNA pol II at both *WEE1* and *ZNF143* promoters. ChIP experiments using anti-CHD5 sera indicate the enrichment of CHD5 at the *WEE1* promoter but not the ZNF143 promoter. As a negative control, ChIP experiments using pre-immune sera (pre-IgG) were performed and show no specific enrichment patterns. Values shown indicate bound v. input signals normalized to a distal intergenic region. (C) As a negative control, ChIP experiments were performed using anti-CHD5 sera and chromatin from KELLY cells (CHD5-negative). No enrichment was observed at the WEE1 promoter using KELLY cells. Error bars represent SD [n = 3].

As mentioned above, CHD5 could either directly regulate the transcription of *WEE1* or could indirectly affect its expression by regulating other transcription factors that, in turn, modulate the expression of *WEE1*. To determine whether *WEE1* is a direct target of CHD5, we performed chromatin immunoprecipitation (ChIP) analyses of CHD5 in PANC-1 cells, which express endogenous CHD5. To identify the region corresponding to the *WEE1* promoter, we performed ChIP using anti-RNA polymerase II (pol II) antibodies, followed by quantitative PCR using sets of primers that span the 5’ end of the *WEE1* locus and the ZNF143 gene, which is approximately 115 kb upstream of *WEE1* (Figure 3A). We observed an enrichment of fragments near the 5’ end of *WEE1 and ZNF143*, indicating that these regions do contain the promoters of these two genes (Figure 3B). We then performed the ChIP analysis using anti-CHD5 antibodies and also observed enrichment of sequences near the promoter of the *WEE1* gene (Figure 3C) but not at regions flanking the promoter or at the ZNF143 promoter. We also did not observe significant enrichment of promoter sequences when pre-immune sera were used, or when anti-CHD5 antibodies were used in KELLY cells (*i.e.*, CHD5-deficient; Figure 3D). The enrichment of CHD5 at the *WEE1* promoter in PANC-1 cells implicates *WEE1* as a direct target for endogenously expressed CHD5.

### A catalytically-inactive mutant CHD5 fails to repress transcription

**Figure 4:**
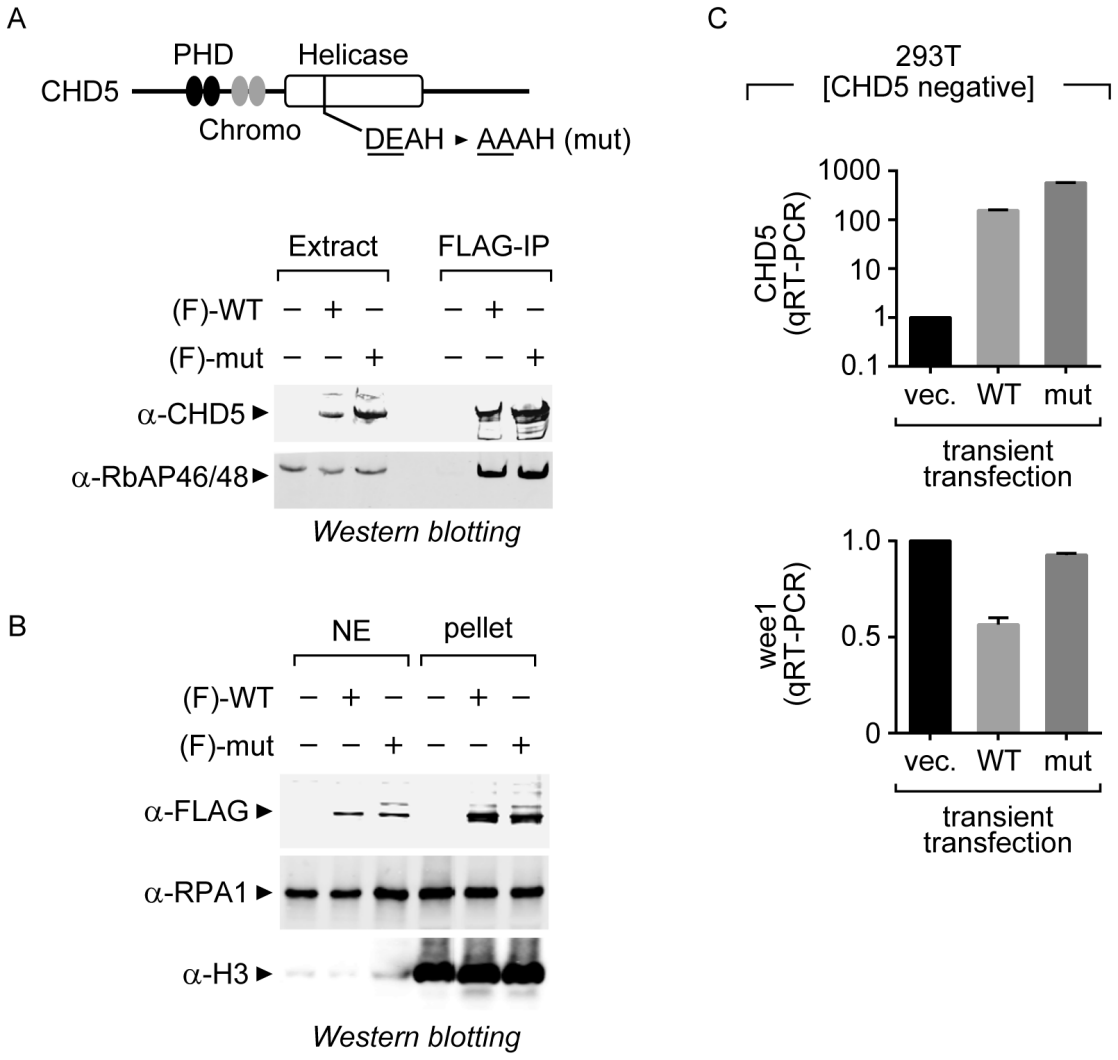
A catalytically inactive mutant CHD5 does not repress transcription. (A) Top, the mutant version of CHD5 (mut CHD5) contains a two-amino acid substitution in the Walker B motif of the ATPase domain. FLAG-tagged [F] wild-type and mut CHD5 expressing plasmids were transiently transfected into 293T-derived cells. Bottom, the CHD5 proteins were immunoprecipitated with anti-FLAG antibodies and the samples analyzed by western blotting against CHD5 or the NuRD subunit RbAP46/48. (B) Soluble, high-salt nuclear extracts or chromatin pellet extracts were prepared from stably transfected PANC-1 cells and used in western blotting assays with antibodies against the FLAG tag, the replication protein A subunit RPA1, or core histone H3. (C) Quantitative RT-PCR analysis of WEE1 transcripts after transfection of wild-type or mut *CHD5*. The expression of wild-type and mut CHD5 were also analyzed using primers against the transfected *CHD5*. The values were normalized to the 5S rRNA housekeeping transcript. Error bars represent SD [n = 3].

CHD5 is part of the SNF2-like family of helicase-related protein and was recently shown to be an ATP-dependent chromatin remodeling factor [4]. To determine whether the remodeling activity of CHD5 is required for its ability to repress transcription, we analyzed a catalytically-inactive mutant version of CHD5 (mut CHD5). The mut CHD5 contains a two amino acid substitution in the conserved Walker B motif (Figure 4A, top), which is required for ATP hydrolysis. We have previously shown that mut CHD5 does not hydrolyze ATP or remodel chromatin [4].

To first determine whether mut CHD5 associates with the NuRD complex, we transiently transfected the HEK293T-derived cells with constructs that express FLAG-tagged copies of wild-type or mut CHD5, and immunoprecipitated the CHD5 proteins using anti-FLAG antibodies (Figure 4A, bottom). We then performed western blotting against one of the NuRD subunits, RbAP46/48. Both wild-type and mut CHD5 proteins interact with RbAP46/48, suggesting that the chromatin remodeling activity of CHD5 is not required to incorporate CHD5 into the complex.

Next, we investigated whether mut CHD5 is able associate with chromatin in cells. In general, CHD5 is poorly extracted from nuclei using traditional high salt (*i.e.*, 0.42 M KCl) extraction methods. In order to fully extract CHD5 from cells, the insoluble material that remains following high salt extraction needs to be homogenized and digested with micrococcal nuclease to release the chromatin-bound proteins. To determine whether mut CHD5 has similar chromatin association properties as wild-type CHD5, we stably transfected PANC-1 cells with the FLAG-tagged wild-type or mut CHD5 constructs and prepared nuclear and solubilized pellet extracts from the cells (Figure 4B). We then performed western blotting of the extracts and found no significant difference in the extractability of wild-type and mut CHD5 proteins. While some wild-type and mut CHD5 proteins were observed in the high-salt soluble extract, most of the wild-type and mut CHD5 protein was in the MNase digested pellet fraction. As controls, we performed western blotting for the replication protein A subunit, RPA1, which is evenly distributed between soluble and pellet extracts, and histone H3, which is almost exclusively found in the pellet fraction. This finding, along with the observation that mut CHD5 is likely incorporated into the NuRD complex, suggests that the two-amino acid substitution in the mut CHD5 protein does not lead to gross changes in its solubility or conformation.

To determine whether the ATPase activity of CHD5 is required to repress transcription of *WEE1*, we transiently transfected HEK293T-derived cells with the either wild-type or mut CHD5 constructs, extracted RNA from the transfected cells, and performed a qRT-PCR analysis as described above (Figure 4C). We measured the levels of CHD5 transcripts from the transfected plasmids, as well as the endogenous levels of *WEE1* mRNA and the 5S rRNA control. We found that, unlike wild-type CHD5, the mut CHD5 was unable to repress transcription of *WEE1*, suggesting that the chromatin remodeling activity is required for the regulation of at least a subset of CHD5-dependent genes. The mechanism by which the ATP-dependent chromatin remodeling activity of CHD5 represses transcription is currently unknown. In general, many chromatin remodeling factors act to increase the accessibility of DNA to DNA-binding proteins. It is possible that the remodeling activity of CHD5 helps load the NuRD cofactors onto the DNA. CHD5 may accomplish this by unwrapping nucleosomes to allow binding of the NuRD subunits that possess DNA binding activities (*e.g.*, MBDs or MTAs) or to expose the underlying histones to the histone binding subunits (*e.g.*, HDACs and RbAP46/48). Another possibility is that the chromatin remodeling activity of CHD5 serves to enhance the binding of CHD5, which serves as a hook to lock the NuRD complex onto chromatin. We have previously shown that the affinity of CHD5 for mononucleosomes is significantly higher in the presence of ATP than in the presence of non-hydrolyzable AMP-PNP, suggesting that CHD5 remains tightly associated with remodeled nucleosomes. Thus far, we have not able to generate viable cell lines that stably express mut CHD5 at levels that are sufficient CHIP analysis and, therefore, we have been unable to determine whether the mut CHD5 binds the *WEE1* promoter.

The fact that the mut CHD5 is no more enriched in the high-salt nuclear extracts than wild-type CHD5 suggests that the activity of CHD5 may not be required for the binding of CHD5-NuRD complexes at every target. The presence of DNA and histone-binding subunits in the NuRD complex may play a major role in binding to most sites across the genome, while the activity of CHD5 may be required for only a subset of loci. If true, this model could shed light on the reason why cells like the precursor cells of the peripheral nervous system need both CHD4-NuRD and CHD5-NuRD complexes. Because the cofactors for CHD4 and CHD5 appear to be identical, the differences in the two complexes may reside exclusively with CHD4 and CHD5. Both CHD4 and CHD5 contain histone-binding chromodomains and PHD fingers, which may target CHD4 and CHD5 to distinct chromatin domains in the cell. Studies on the histone-binding properties of the chromodomains and PHD fingers of CHD4 and CHD5 have not shown clear differences between them but those studies are ongoing (*e.g.*, see [40]–[42]). It is also possible that CHD4 and CHD5 have distinct remodeling activities and that some feature of neural-cell chromatin is resistant to the remodeling activity of CHD4. This model is consistent with our previous biochemical analysis that shows that, in vitro, CHD4 does not possess the robust nucleosome unwrapping activity observed with CHD5 [4]. This is true despite the fact that CHD4 possesses a high specific activity in other remodeling assays [4]. Thus, the remodeling activity of CHD5 may facilitate the binding of the NuRD complex to sites with unusual structural features or patterns of histone and DNA modification.

In addition to demonstrating that CHD5-NuRD complexes exist in human cells and that CHD5 represses transcription, we also identified *WEE1* as a CHD5-dependent gene. Whether or not the tumor suppressor role of CHD5 is linked to *WEE1* expression is not fully clear. WEE1 acts to inhibit the cell cycle and derepression of *WEE1* expression upon loss of CHD5 should enhance the G2/M checkpoint. However, the fact that WEE1 appears to act as an oncogene in several cancers, including neuroblastoma, suggests that restricting entry into mitosis is an important step during tumor progression. Slowing entry into mitosis likely gives the dividing cells adequate time to complete S-phase and repair any DNA lesions [39]. This regulation becomes increasingly important when cells acquire MYCN amplification, which accelerates cell growth [43]. In normal cells, CHD5 acts as a tumor suppressor that is dependent on other pathways involved in cell-growth, such as p16 and p19 [7]. For example, induced expression of CHD5 in normal MEFs leads to a reduction in cell proliferation [21]. Whether CHD5 is required to regulate the levels of *WEE1* in the context of normal cell growth is not clear. On the other hand, loss of CHD5 in cancer cells may provide a selective advantage by allowing the cells to elevate their levels of *WEE1* expression. The fact that neuroblastoma cells have elevated levels of WEE1 and are sensitive to WEE1 inhibitors [38] supports this model. Expression of CHD5 in the neuroblastoma cells was previously shown to limit cell growth, although the effects were modest [21]. Similarly, we found that transient expression of CHD5 in KELLY cells can combine with a WEE1 inhibitor to further limit cell viability (Figure S4).

It is surprising that in the context of cells that have lost CHD5, the presence of the related factor, CHD4 does not appear to adequately compensate for the loss of CHD5. While it is possible that CHD4 and CHD5 have some overlapping activities, the presence of CHD4 in neuroblastoma cells suggests that the overlap is limited. It also implies at least some separation of function of the CHD4 and CHD5-containing NuRD complexes.

## Supporting Information

Figure S1 **Mass spectrometry results of the factors present in the CHD5-containing complex that was purified by tandem affinity purification from HeLa cells.** The number of unique peptides for each factor is listed.
(TIF)

Figure S2 **Top hits from the microarray analysis of KELLY cells transiently transfected with vector or a CHD5 cDNA.** The fold change represents the signal from the CHD5 transfected cells divided by the signal from the cells transfected with the vector. CHD5 and *WEE1* (highlighted in gray) were validated by qRT-PCR. The raw data are accessible at NCBI [GEO Accession: GSE59899].
(TIF)

Figure S3 (A) Transient expression of CHD5 at low levels in Kelly cells does not significantly affect CHD4 protein or transcript levels. (B) siRNA-mediated knockdown of CHD5 in PANC-1 cells does not affect CHD4 protein levels. The replication protein A subunit, RPA2 was used as a control.
(TIF)

Figure S4 **Combining transient expression of CHD5 in KELLY cells with a** ***WEE1*** **inhibitor reduces cell growth.** KELLY cells were transiently transfected with vector or the CHD5 cDNA and incubated with a *WEE1* inhibitor. The relative cell count was determined by measuring the cell viability at the indicated days divided by the starting cell viability at day 0. Data are represent mean and SD [n = 3].
(TIF)

Table S1 **Sequences of the primers used in the ChIP analyses.**
(DOC)
