# Supplementary figures and images for: The Chromatin Remodeling Factor CHD5 Is a Transcriptional Repressor of WEE1
Source: PLoS One. 2014 Sep 23;9(9):e108066. doi: 10.1371/journal.pone.0108066 (PMC4172601; doi:10.1371/journal.pone.0108066)

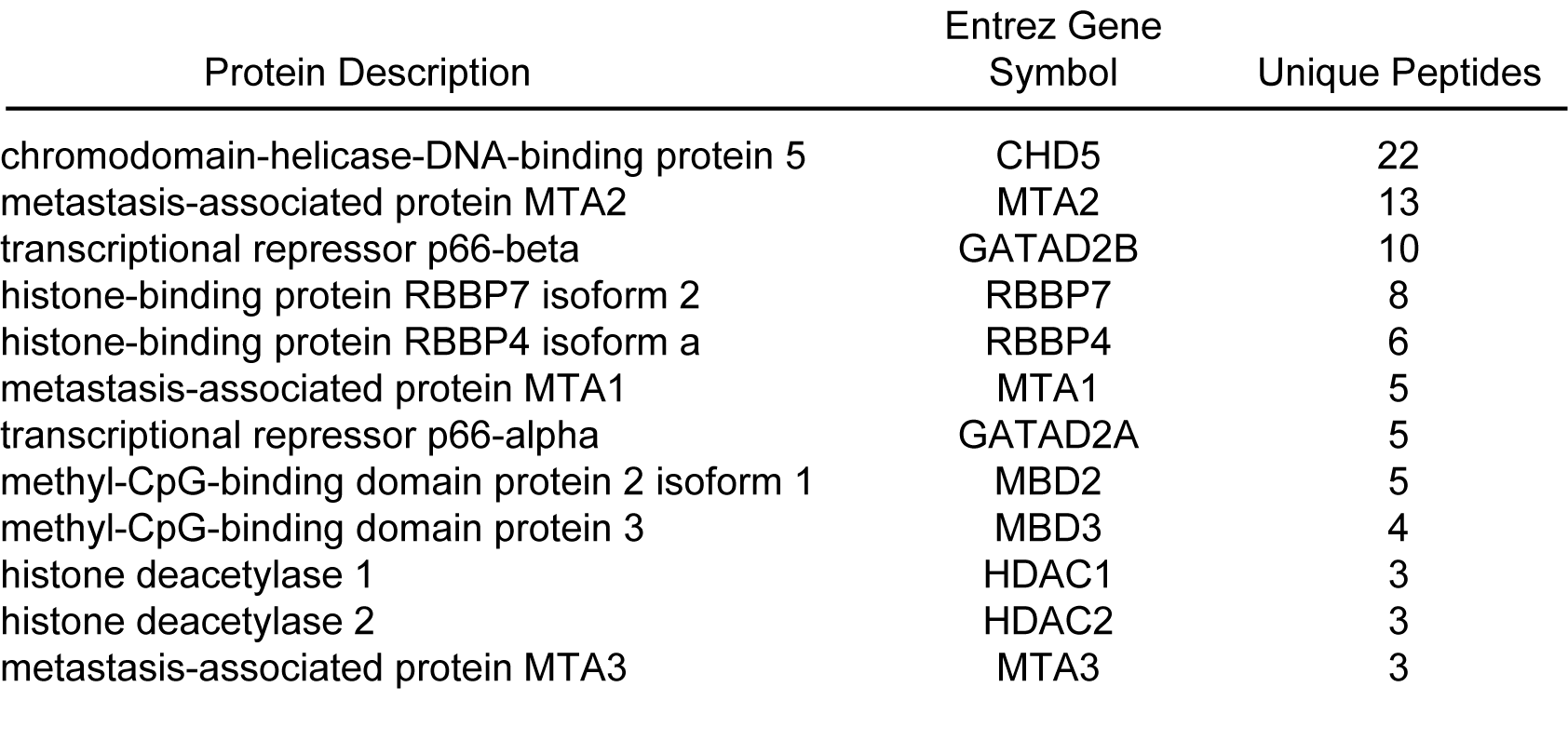

Supplement: Figure S1 — Mass spectrometry results of the factors present in the CHD5-containing complex that was purified by tandem affinity purification from HeLa cells. The number of unique peptides for each factor is listed. (TIF) [file pone.0108066.s001.tif]

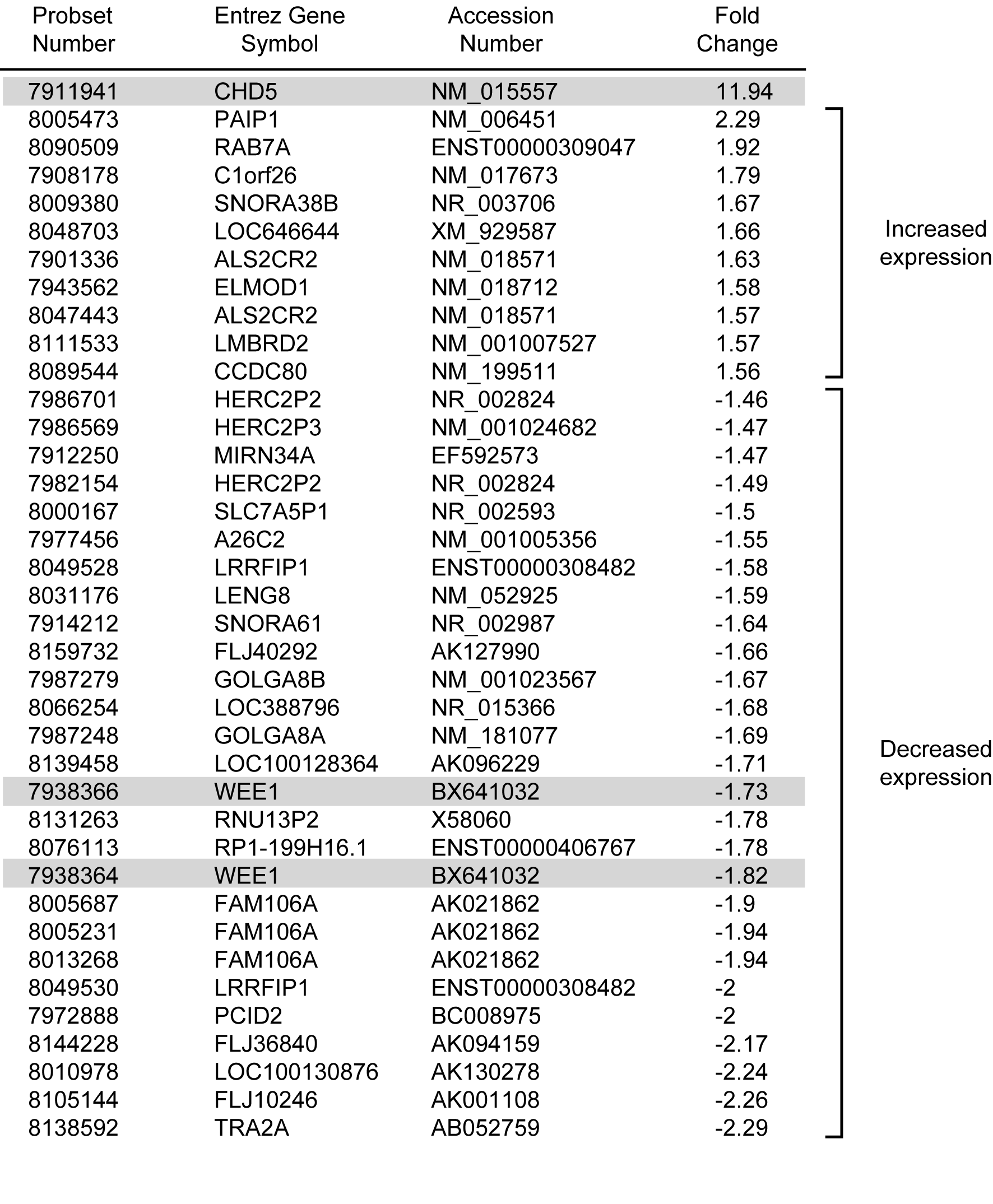

Supplement: Figure S2 — Top hits from the microarray analysis of KELLY cells transiently transfected with vector or a CHD5 cDNA. The fold change represents the signal from the CHD5 transfected cells divided by the signal from the cells transfected with the vector. CHD5 and WEE1 (highlighted in gray) were validated by qRT-PCR. The raw data are accessible at NCBI [GEO Accession: GSE59899]. (TIF) [file pone.0108066.s002.tif]

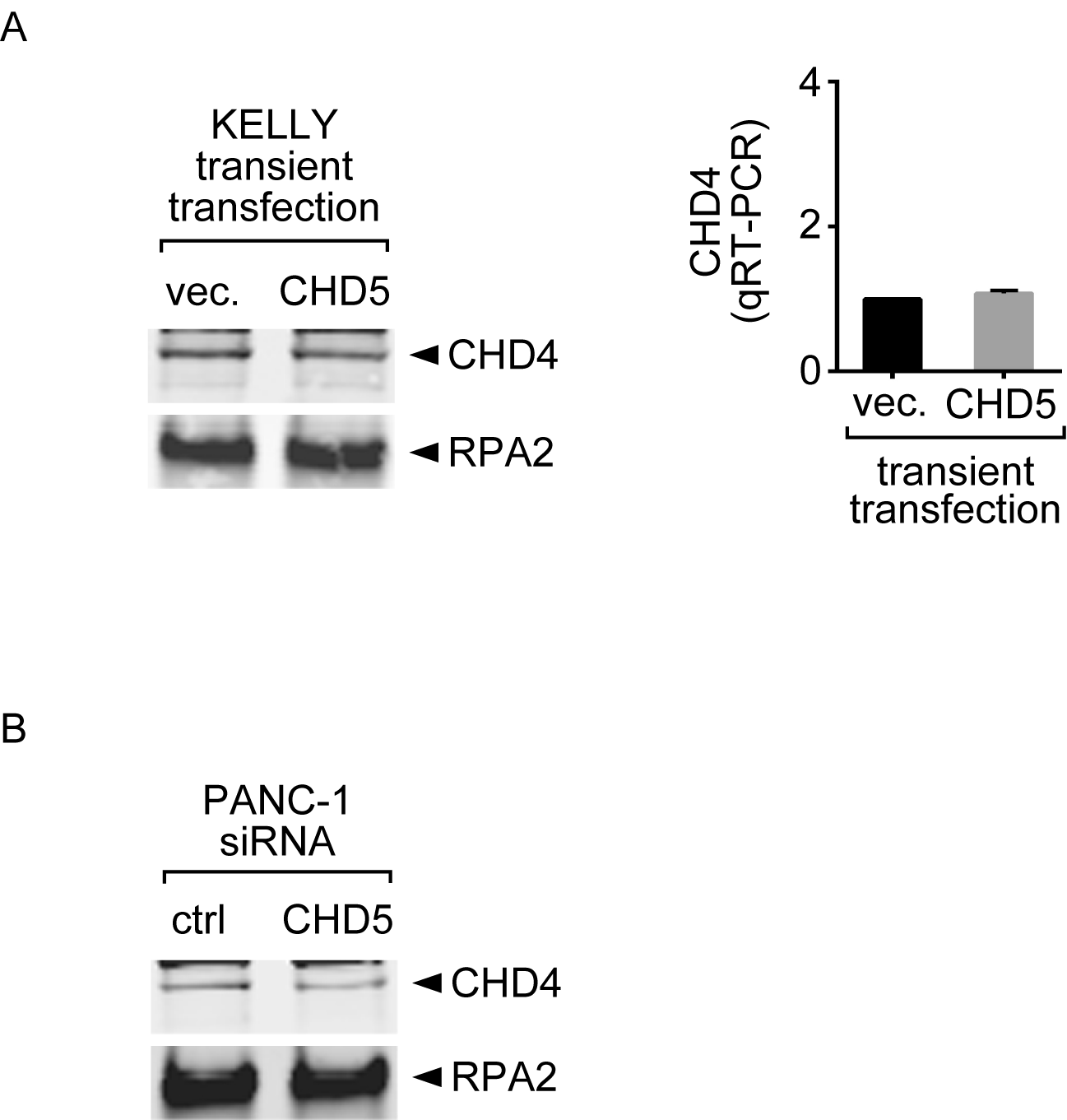

Supplement: Figure S3 — (A) Transient expression of CHD5 at low levels in Kelly cells does not significantly affect CHD4 protein or transcript levels. (B) siRNA-mediated knockdown of CHD5 in PANC-1 cells does not affect CHD4 protein levels. The replication protein A subunit, RPA2 was used as a control. (TIF) [file pone.0108066.s003.tif]

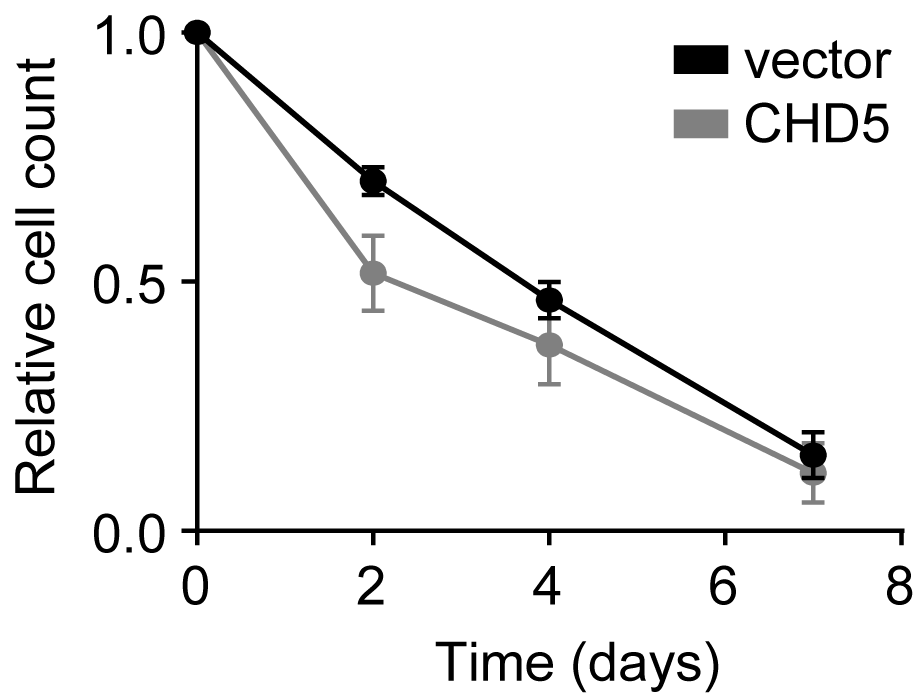

Supplement: Figure S4 — Combining transient expression of CHD5 in KELLY cells with a WEE1 inhibitor reduces cell growth. KELLY cells were transiently transfected with vector or the CHD5 cDNA and incubated with a WEE1 inhibitor. The relative cell count was determined by measuring the cell viability at the indicated days divided by the starting cell viability at day 0. Data are represent mean and SD [n = 3]. (TIF) [file pone.0108066.s004.tif]
