# Supplementary material for: The Chromatin Remodeling Factor CHD5 Is a Transcriptional Repressor of WEE1
Source: PLoS One. 2014 Sep 23;9(9):e108066. doi: 10.1371/journal.pone.0108066 (PMC4172601; doi:10.1371/journal.pone.0108066)
Supplement: Table S1 — Sequences of the primers used in the ChIP analyses. (DOC) [file pone.0108066.s005.doc]

ChIP primers:

| Promoter (set 1) | For: 5’– CTTTTGCCCCTCCCAGAAG –3’ |
| --- | --- |
| Promoter (set 1) | Rev: 5’– CAGCTCCGGGTTTGAAAAAA –3’ |
| Promoter (set 2) | For: 5’– ATTCGGTCCCTGGCTCTT –3’ |
| Promoter (set 2) | Rev: 5’– AGAGAGCTGGATTCGCCTAA –3’ |
| 1.5kb upstream | For: 5’– AACCCATGACAACTGTTCAACAA –3’ |
| 1.5kb upstream | Rev: 5’– GCACACTCTGTGAATTCAGGACAT –3’ |
| 2kb downstream | For: 5’– TTTGTCATCAGCCTAGGCAATCT –3’ |
| 2kb downstream | Rev: 5’– TTGAGCAAGTCGGCCAATAAA –3’ |
| 5kb downstream | For: 5’– CCAGGGTTCAAGCGATTCTC –3’ |
| 5kb downstream | Rev: 5’– GGCGCGCATCTGTAATCC –3’ |
| 8kb upstream | For: 5’– TGACCCTGCCTTCATCAAATCT –3’ |
| 8kb upstream | Rev: 5’– CCTACCCCAGTAAGAAGGGCATA –3’ |
| ZNF143 promoter (set 1) | For: 5’– GTTTAGAAAGGGCTGGGTTAAGA –3’ |
| ZNF143 promoter (set 1) | Rev: 5’– GCATTCAGTCTGGTTCCCTAAA –3’ |
| ZNF143 promoter (set 2) | For: 5’– CAGGTCTCTCTTTCTGTCCTATTT –3’ |
| ZNF143 promoter (set 2) | Rev: 5’– CAGACCAGAAGGGCTTTGT –3’ |
| Distal (chr1) Control | For: 5’– GCAAGTAGAATTAGGCAGGGTTGT –3’ |
| Distal (chr1) Control | Rev: 5’– AGGTATCTGTGTTTAGCCCTGCAT –3’ |
